# Burden of Care for Children with Bronchiectasis from Parents/Carers Perspective

**DOI:** 10.3390/jcm10245856

**Published:** 2021-12-14

**Authors:** Julie M. Marchant, Anne L. Cook, Jack Roberts, Stephanie T. Yerkovich, Vikas Goyal, Daniel Arnold, Hannah E. O’Farrell, Anne B. Chang

**Affiliations:** 1 Australian Centre for Health Services Innovation @ Centre for Healthcare Transformation, Queensland University of Technology, Brisbane, QLD 4059, Australia; anne.cook@qut.edu.au (A.L.C.); jack.roberts@qut.edu.au (J.R.); stephanie.yerkovich@menzies.edu.au (S.T.Y.); drvikasgoyal@gmail.com (V.G.); d5.arnold@qut.edu.au (D.A.); hannah.ofarrell@menzies.edu.au (H.E.O.); anne.chang@menzies.edu.au (A.B.C.); 2 NHMRC Centre for Research Excellence in Paediatric Bronchiectasis (AusBREATHE), Queensland Children’s Hospital, Brisbane, QLD 4101, Australia; 3 Department of Respiratory and Sleep Medicine, Queensland Children’s Hospital, Brisbane, QLD 4101, Australia; 4 Menzies School of Health Research, Charles Darwin University, Darwin, NT 0810, Australia; 5 School of Medicine, University of Queensland, Brisbane, QLD 4072, Australia

**Keywords:** bronchiectasis, paediatrics, burden, quality of life, education, knowledge, management plan

## Abstract

Bronchiectasis is a neglected chronic respiratory condition. In children optimal appropriate management can halt the disease process, and in some cases reverse the radiological abnormality. This requires many facets, including parental/carer bronchiectasis-specific knowledge, for which there is currently no such published data. Further, the importance of patient voices in guiding clinical research is becoming increasingly appreciated. To address these issues, we aimed to describe the voices of parents of children with bronchiectasis relating to (a) burden of illness and quality of life (QoL), (b) their major worries/concerns and (c) understanding/management of exacerbations. The parents of 152 children with bronchiectasis (median age = 5.8 years, range 3.5–8.4) recruited from the Queensland Children’s Hospital (Australia) completed questionnaires, including a parent-proxy cough-specific QoL. We found that parents of children with bronchiectasis had impaired QoL (median 4.38, range 3.13–5.63) and a high disease burden with median 7.0 (range 4.0–10.0) doctor visits in 12-months. Parental knowledge varied with only 41% understanding appropriate management of an exacerbation. The highest worry/concern expressed were long-term effects (*n* = 42, 29.8%) and perceived declining health (*n* = 36, 25.5%). Our study has highlighted the need for improved education, high parental burden and areas of concern/worry which may inform development of a bronchiectasis-specific paediatric QoL tool.

## 1. Introduction

Bronchiectasis is a heterogenous condition of persistent/chronic or recurrent wet cough, usually with airway infection and inflammation, and abnormal dilatation of the airway on chest computed tomography scans [1]. It is a major contributor to chronic lung morbidity and the prevalence of bronchiectasis is increasing worldwide, which is likely from a combination of increased recognition and increased incidence [2]. The disease is characterised by recurrent respiratory exacerbations with some resulting in hospitalisations [3]. Whilst bronchiectasis was previously thought to be irreversible, it has now been shown that when optimally managed, the progression of bronchiectasis can be halted in most, and the radiological airway abnormality can even reverse in children [3], and some adults [4,5]. Thus, it is crucial that children receive optimal care. To do so, we need to hear and heed the voices from the patients and parents of children, and it is increasingly appreciated that the knowledge they provide is very valuable in both the clinical and research contexts [6]. Further, the importance of the patient and caregivers’ perspective has been identified as imperative in guiding future research [7,8]. Yet there is a relative paucity of data on general concerns/worries of parents/carers (hereafter referred to as parents) of children with bronchiectasis or their current knowledge base, especially relating to respiratory exacerbations. Exacerbations are particularly important in paediatric bronchiectasis from both parents’ and clinical perspectives. In the recent European Lung Foundation (ELF)-led survey, having an action management plan was high on their ‘wish list’ [9]. From the clinical perspective, current data have shown that exacerbations are particularly important as they are associated with increased parental psychological stress [10], impaired quality of life (QoL) [10], lung function decline (−1.9 FEV_1_% predicted per hospitalised exacerbation) [11] and substantial healthcare costs (costing the healthcare system ~AUD 33,000 for each hospitalisation) [12].

The recommendations from the recent European Respiratory Society (ERS) clinical practice guideline for managing children/adolescents with bronchiectasis include early and appropriate management using a multidisciplinary approach, including that for exacerbations [13]. To adhere to these guidelines, parents and caregivers need education and understanding about bronchiectasis symptoms and the management of exacerbations. Thus, understanding parental concerns/worries and having data on their knowledge related to the identification and management of exacerbations is imperative. It will likely help guide clinicians to educate parents, develop relevant resources and guide future research, including that related to development of a paediatric bronchiectasis QoL tool.

Our study aimed to describe the voices of parents of children with bronchiectasis relating to (a) the burden of illness and quality of life (QoL), (b) their major worries/concerns and (c) understanding and management of exacerbations. We hypothesised that parents of children with bronchiectasis have common worries/concern and variable knowledge on how they identify and/or self-manage respiratory exacerbations.

## 2. Materials and Methods

### 2.1. Study Setting and Design

This study was planned as sub-study at the largest recruitment site (Queensland Children’s Hospital, Brisbane) of a current multicentre randomised controlled trial (RCT) being undertaken at 3 sites (Brisbane (Queensland), Darwin (Northern Territory), and Perth (Western Australia)) on the utility of a personalised bronchiectasis action management plan [14]. The Human Research Ethics Committees of Queensland Children’s Hospital, Brisbane (HREC/18/QCHQ/45348)) have approved this study.

### 2.2. Study Population

The inclusion criteria were children with bronchiectasis: (1) aged <19 years and (2) had ≥2 non-scheduled doctor visits or respiratory exacerbations in the previous 18 months. They were recruited from both outpatient clinics and inpatient wards and could be in any state of their bronchiectasis (i.e., stable or exacerbation). We excluded children with cystic fibrosis and parents who could not speak or read English. All parents provided written informed consent. Participants were recruited from February 2019 to July 2020.

### 2.3. Study Methods

At enrolment, baseline data were collected from parents and medical records. This data included demographic data (such as age, gender, ethnicity) and healthcare burden information such as prior cough and illness frequency, number of acute respiratory exacerbations and hospitalizations. Parents also completed a validated 8-item parent-proxy chronic cough QoL (PC-QoL-8) [15]. In this PC-QoL, parents answer 8 questions, and a summary score (average) is attained with a range between 1 (most impact i.e., poorest QoL) and 7 (no impact i.e., best QoL). The Pc-QoL questions are cough-specific and ask during the past week how often: (i) Did you feel upset because of your child’s cough? (ii) Were you awakened during the night because of your child’s cough? (iii) Did you feel helpless because of your child’s cough? (iv) Did you feel scared because of your child’s cough? (v) Did you feel you were being overprotective because of your child’s cough? (vi) Were you worried about your child being able to lead a normal life? (vii) Were you worried about leaving your child with others because of his/her cough? (viii) Were you worried about your child not sleeping well because of the cough? Parents rank their answers on a scale from 1 (all of the time/very, very worried) to 7 (none of the time/not worried) and their 8 answers are averaged for a final score [15].

Parents also answered a questionnaire that consisted of 5 questions: (i) What are five things that worry or bother you most about your child’s bronchiectasis? (ii) What do you do when your child has a wet cough/exacerbation of their bronchiectasis? (iii) How long would you wait when your child has a wet cough to seek help or start treatment? (iv) When would you see a doctor? (v) Have you ever had written instructions for managing your child’s bronchiectasis before? All data was de-identified and maintained in a password protected database.

### 2.4. Statistical Analysis

Data were expressed as medians and interquartile range (IQR) or counts (%). The software Statistical Package for the Social Sciences (SPSS) 27.0 (SPSS Inc., Chicago, IL, USA) was used for analysis.

## 3. Results

### 3.1. Demographic Data

The median age of the 152 children recruited was 5.8 years (IQR 3.5, 8.4) and 57.2% (*n* = 87) were male (Table 1). The most common cause of bronchiectasis was post infectious. While most of the children were in stable state 28.3% (*n* = 43) were unwell with an exacerbation at the time of enrolment, and of these 35 children (23%) had their wet cough heard either spontaneously or ‘on request’ at enrolment.

### 3.2. Burden of Illness

The median PC-QoL-8 score of 4.38 (IQR 3.13, 5.63) indicated poor QoL in our cohort, and it was worse in those in exacerbation state at time of enrolment (Table 2). Many of the parents (77.6%, *n* = 118) reported at least one episode of 4 weeks of daily chronic wet cough in the previous 12-month period. Almost half the cohort (47.4%, *n* = 72) had been hospitalised for an exacerbation in the prior 2-year period and 69% (*n* = 105) had been hospitalised ever for an exacerbation. Table 2 illustrates other aspects of the disease burden, including frequent doctor visits, over the previous 12-month period.

### 3.3. Top Worries/Concerns of Parents

The concerns of parents of the children with bronchiectasis when asked “What are five things that most worry or concern you about your child having bronchiectasis?” are listed in Table 3 as percentage of parents who voiced this as one of their five greatest concerns. A total of 141 parents answered this question and provided 562 items. Other items not listed in Table 3 which were identified at lower frequency (but ≥ 5 times) were: leaving child with others (*n* = 9, 6.4%), impact on family (*n* = 9, 6.4%), child feeling unwell (*n* = 9, 6.4%), child choking (*n* = 8, 5.7%), ability to manage and support child’s health (*n* = 7, 5.0%), life expectancy (*n* = 6) and anxiety/self-esteem in their child (*n* = 5).

### 3.4. Knowledge Relating to Bronchiectasis Exacerbations

**Figure 1:**
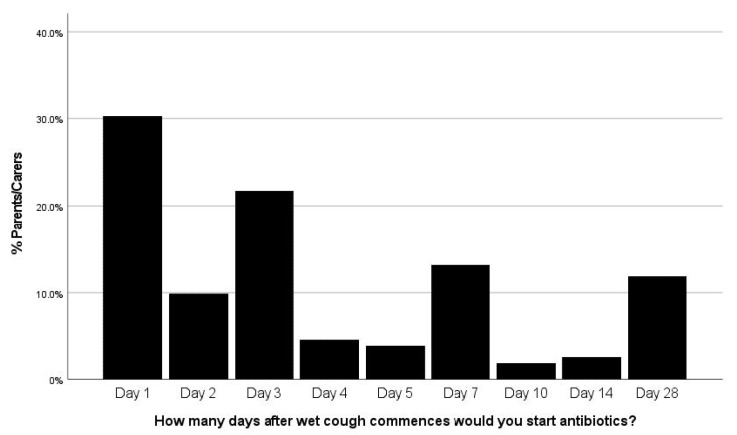
Knowledge of appropriate antibiotic usage in bronchiectasis parent group. What day after wet cough started would they commence antibiotics? (*n* = 152).

There was a large variability in the knowledge-base of the parents. Most had never received a formal written action plan from a doctor (*n* = 121, 79.6%) and very few would self-manage an exacerbation routinely (*n* = 12, 7.9%). Figure 1 shows when parents would start antibiotics after the beginning of a wet cough which varied from Day 1 to Day 28, with a large proportion starting on the first day of wet cough (*n* = 46; 30.3%) or after 28 days of wet cough (*n* = 18; 11.8%). Less than half the participants (*n* = 62; 40.8%) had knowledge of the appropriate management of an exacerbation (defined as commencing an antibiotic and increasing frequency of airway clearance therapy).

## 4. Discussion

In our cohort of children with bronchiectasis, their parents reported impaired QoL and a high illness burden with a large number of respiratory exacerbations, medications, hospitalisations, and doctors’ visits. There was substantial variability of bronchiectasis knowledge relating to exacerbations despite the majority having had the diagnosis for over 12 months and being cared for in a tertiary paediatric hospital. The five leading parental worries/concerns were the impact of the illness on their child’s future health and ability to lead a normal life, the possibility of declining health, the child’s cough, the impact of bronchiectasis on the child’s play and development and the parents’ lack of sleep/tiredness.

Our study has provided novel data in several aspects. Firstly, it is the first to detail the large variability of knowledge of bronchiectasis within a paediatric cohort, with less than half of our parents having knowledge of appropriate management of a respiratory exacerbation and most (79.6%) having never been provided with a written plan from their doctors. Secondly, it has clearly documented the areas of worry and concern that contribute to poorer QoL in children with bronchiectasis and their parents within a large cohort from a single centre.

The illness burden in our cohort was considerable with an average of seven doctors’ visits each year for their bronchiectasis and a median of four non-hospitalised exacerbations in the prior 24 months. These finding are comparable to previous studies [16,17,18]. Additionally, the illness burden is reflected in the reported frequent chronic (>4 weeks) wet cough episodes (over three quarters in 12 months prior), and high use of antibiotics with on average two (range one to four) scripts filled for treatment of exacerbations. A previous multicentre study on healthcare use in exacerbations reported antibiotic use of 50 per 100 child months which is higher than that in our cohort (equates to 18.4 per child month) but as they included azithromycin prescriptions, which are not exacerbation related and used in a large proportion of children, this is likely comparable [18]. They further stated approximately one third of their cohort had a prescription or antibiotics at home to use at the time of an exacerbation [18], which is in keeping with our findings of 41% of parents having appropriate knowledge of exacerbation treatment.

Our study showed a significantly impaired PC-QoL in children with bronchiectasis. A comparison of PC-QoL scores in children with chronic cough of other causes in a large multi-centered study found no significant difference (*p* = 0.42) between cough-specific QoL scores in children who presented with chronic cough and were subsequently diagnosed with various conditions, including protracted bacterial bronchitis, asthma or bronchiectasis [19]. The QoL score was not influenced by etiology nor age nor duration of cough. Their cohort (*n* = 346) had median PC-QoL scores of 3.8 (IQR 1.9, 5.0) comparable to our findings [19]. They also highlight that QoL in their cohort (also measured by a generic pediatric QoL, PedsQL) was comparable to chronic conditions such as cardiac disease and diabetes [19]. In addition, it has been shown in a cohort of children with chronic cough (*n* = 272) that when the cough resolves the PC-QoL scores improve significantly from median of 3.6 (IQR 2.7, 4.9) to 6.5 (IQR 5.2, 7) which is approaching population normal values [20].

Many studies have shown children (and their parents) with bronchiectasis have an impaired QoL, which worsens during an acute exacerbation [10,18]. The PC-QoL-8 in our cohort indicated a reduced QoL similar to those previously reported when in an acute exacerbation [10,17], although only 28.3% had a current exacerbation at recruitment. Unlike these studies which recruited and assessed PC-QoL in both stable state and exacerbation state our study enrolled children at various times (steady state and exacerbation) and from both outpatient clinics and inpatient hospitalisations, which may explain the poorer QoL compared to children who were currently stable. In addition, our inclusion criteria stated children had two or more non-scheduled doctor visits or respiratory exacerbations in the previous 18 months hence suggesting they had more severe disease then children with bronchiectasis not having symptoms at this frequency. Our study PC-QoL-8 (median 4.38, range 3.13, 5.63) is comparable to exacerbation state in previous intervention studies [16,17]. It is also possible this illustrates the fact there are many other contributors to QoL in bronchiectasis, other than cough and questions contributing to the PC-QoL-8, which are relevant to the disease burden, yet the cough-specific PC-QoL-8 tool is most frequently used to define QoL in paediatric bronchiectasis. The need for a disease-specific tool was addressed in adults many years ago with the development of an adult bronchiectasis-specific QoL tool [21]. As adult tools are not appropriate for use in children [22], there is a need for a paediatric bronchiectasis-specific QoL tool.

The degree of variability of bronchiectasis knowledge within the parent cohort was one of the most novel findings of our study. Despite being cared for in a hospital by respiratory specialists and the majority having had a bronchiectasis diagnosis for over one year, parental knowledge on management of respiratory exacerbations was poor. Less than half of the parents (41%) articulated that appropriate management of a respiratory exacerbation, as defined by recent ERS paediatric guidelines [13], was the early use of antibiotics and increasing frequency of airway clearance techniques. The variability in knowledge of when to start antibiotics for wet cough was large. Further, only 8% of parents felt they would self-manage a respiratory exacerbation. A recent novel study which utilised social media listening found exacerbations and antibiotic use were common concerns found in patients with bronchiectasis, confirming our findings in children [23]. The management of respiratory exacerbations appropriately is an imperative component of optimising clinical care for children with bronchiectasis as it can reduce disease severity [3]. In addition, if treated early and appropriately the disease can be reversed in some children [3,13,24]. Our results show that education surrounding acute respiratory exacerbations and self-management is lacking in parents of children with bronchiectasis. An educational tool which has been shown to work effectively in management of acute exacerbations and is recommended in other chronic respiratory diseases, such as asthma, is a personalised action plan [25].

To date there are no studies evaluating an action management plan in children with bronchiectasis [26]. The ERS and ELF have highlighted the need for an action management plan for respiratory exacerbations amongst the highest clinical needs for parents of children with bronchiectasis [7]. Another top clinical priority of this taskforce document was “good communication between healthcare professionals and each person with bronchiectasis” [7]. As over three quarters of parents had never had a written management plan from their doctor our research further supports this need in another cohort of parents of children with bronchiectasis.

The patient and caregiver perspective in health-related QoL is now identified as best practice [8,27]. “Impact on adult life” and “ongoing declining health” were the most common concerns and worries identified by parents of children with bronchiectasis. Other common concerns included use of antibiotics, lack of sleep, ability to play sport and lack of information about exacerbations. It is known that acute respiratory exacerbations of bronchiectasis in children are associated with a poorer QoL and increased psychological stress [10]. Our results explain in children, through the perspective of their parents, concerns and worries that are contributing to this stress and impaired QoL. It has identified statements which could be used as a basis for development of a bronchiectasis-specific QoL tool, many of which are not included in the tool currently used to measure QoL in bronchiectasis intervention trials such as the PC-QoL-8 used in our study [15]. It can also be seen that many of these areas of worry could be alleviated with appropriate education enabling parents to self-manage acute respiratory exacerbations beyond the very low rates found in our study.

The major limitation of this study is that it is a single centre study and the view reflected may not be those of a different healthcare setting. It is also a narrative descriptive study which lacks a control group. However, our population demographics are similar to published paediatric cohorts [17]. In addition, as it was undertaken in a large tertiary hospital with a significant bronchiectasis population one would expect the population to be better educated and informed then other cohorts. A further limitation is that all healthcare usage was parent-reported and as such may be subject to recall bias. Further, our inclusion criteria were children with bronchiectasis who had ≥2 non-scheduled doctor visits or respiratory exacerbations in the previous 18 months therefore excluding those with very mild disease. Finally, we did not collect data on religious, economic or educational background of parents which may impact parental burden.

Overall, our study has highlighted the ongoing high burden of care for parents of children with bronchiectasis and identified areas of parental concern. Parental education within a multi-disciplinary clinic structure by health professionals addressing these areas when seeing children with bronchiectasis and their parents is needed. It has illustrated the clinical need for a bronchiectasis action management plan to inform and educate parents on management of exacerbations. Finally, it has highlighted that children with bronchiectasis have areas of function that make an important contribution to QoL, in addition to cough, and hence a bronchiectasis-specific QoL tool is needed.

## Figures and Tables

**Table 1:** Characteristics of 152 children with bronchiectasis.

Characteristic	*n* (%) or Median (IQR)
Male (%)	87 (57.2%)
Age at enrolment (median years, IQR)	5.8 (IQR 3.5, 8.4)
New diagnosis (within 12 months)	38 (25.0%)
Ethnicity	
First nations	15 (9.9%)
Caucasian/other	137 (90.1%)
Cause of bronchiectasis	
Post infectious	91 (59.9%)
Idiopathic	42 (27.6%)
Aspiration	10 (6.6%)
Primary ciliary dyskinesia	5 (3.3%)
Primary immunodeficiency	1 (0.7%)
Missing/other	3 (2.0%)
Number of affected lobes in lungs	2.0 (1.0, 4.0)
Smoke exposure	36 (23.7%)

**Table 2:** Burden of illness (*n* = 152).

	Median (IQR)
PC-QoL-8 ^1^ score on enrolment for cohort	4.38 (3.13, 5.63)
Exacerbation state (*n* = 43)	3.38 (2.63, 5.19)
Stable state (*n* = 109)	4.50 (3.38, 5.69)
Number of hospitalisations post-diagnosis for exacerbations	1.0 (1.0, 4.0)
Non-hospitalised exacerbations requiring antibiotics in last 2 years	4.0 (2.0, 6.0)
Total doctor visits for bronchiectasis in last 12 months	7.0 (4.0, 10.0)
Specialist visits	3.0 (2.0, 4.0)
Emergency department visits	0.0 (0.0, 1.0)
Primary care doctor visits	3.0 (1.0, 6.0)
Antibiotic scripts filled for exacerbations in the last 12 months	2.0 (1.0, 4.0)

^1^ PC-QoL-8 = parent-proxy chronic cough quality of life.

**Table 3:** Greatest concerns and worries voiced by parents of children with bronchiectasis ^1^.

Concern/Worry	*n* (%), Total *n* = 141
Impact on his/her adult life in future, long term effects, “normal life”	42 (29.8%)
Ongoing declining health	36 (25.5%)
The cough	35 (24.8%)
Impact on his/her life now as child: play, development	34 (24.1%)
Lack of sleep/being tired	34 (24.1%)
Concern over aspects of antibiotic use	32 (22.7%)
Missing school or daycare	25 (17.7%)
Breathing difficulties/shortness of breath	23 (16.3%)
Ability to exercise/play sport	21 (14.9%)
Lung damage/lung function	17 (12.1%)
Medications	17 (12.1%)
Lack of information/education about bronchiectasis/needing plan	16 (11.3%)
Exacerbations	16 (11.3%)
Unable to plan, e.g., holidays	16 (11.3%)
Social life/missing important events	15 (10.6%)
Judged by others/Perception of the cough by others	14 (9.9%)
Time off work	14 (9.9%)
Frustration with doctors understanding of bronchiectasis	13 (9.2%)
Quality of life	13 (9.2%)
Hospital admissions	13 (9.2%)
Staying away from others with respiratory infections	11 (7.8%)
Financial implications	10 (7.1%)

^1^ Answers from parents to “What are five things that most worry or concern you about your child having bronchiectasis?”.

## Data Availability

The data are available from corresponding author on request.
